# Electrochemical Microsensors for the Detection of Cadmium(II) and Lead(II) Ions in Plants

**DOI:** 10.3390/s100605308

**Published:** 2010-05-27

**Authors:** Olga Krystofova, Libuse Trnkova, Vojtech Adam, Josef Zehnalek, Jaromir Hubalek, Petr Babula, Rene Kizek

**Affiliations:** 1 Department of Chemistry and Biochemistry, Faculty of Agronomy, Mendel University in Brno, Zemedelska 1, CZ-613 00 Brno, Czech Republic; E-Mails: olga.krystofova@seznam.cz (O.K.); ilabo@seznam.cz (V.A.); josef.zehnalek@seznam.cz (J.Z.); 2 Department of Chemistry, Faculty of Science, Masaryk University, Kotlarska 2, CZ-611 37 Brno, Czech Republic; E-Mail: libuse@chemi.muni.cz (L.T.); 3 Research Centre for Environmental Chemistry and Ecotoxicology, Faculty of Science, Masaryk University, Kotlarska 2, CZ-611 37 Brno, Czech Republic; 4 Department of Microelectronics, Faculty of Electrical Engineering and Communication, Brno University of Technology, Udolni 53, CZ-602 00 Brno, Czech Republic; E-Mail: hubalek@feec.vutbr.cz (J.H.); 5 Department of Natural Drugs, Faculty of Pharmacy, University of Veterinary and Pharmaceutical Sciences Brno, Palackeho 1-3, CZ-612 42 Brno, Czech Republic; E-Mail: petr_babula@email.cz (P.B.)

**Keywords:** heavy metals, lead, cadmium, miniaturization, screen printed electrode, amperometry, voltammetry, plant, maize, sunflower, water

## Abstract

Routine determination of trace metals in complex media is still a difficult task for many analytical instruments. The aim of this work was to compare three electro-chemical instruments [a standard potentiostat (Autolab), a commercially available miniaturized potentiostat (PalmSens) and a homemade micropotentiostat] for easy-to-use and sensitive determination of cadmium(II) and lead(II) ions. The lowest detection limits (hundreds of pM) for both metals was achieved by using of the standard potentiostat, followed by the miniaturized potentiostat (tens of nM) and the homemade instrument (hundreds of nM). Nevertheless, all potentiostats were sensitive enough to evaluate contamination of the environment, because the environmental limits for both metals are higher than detection limits of the instruments. Further, we tested all used potentiostats and working electrodes on analysis of environmental samples (rainwater, flour and plant extract) with artificially added cadmium(II) and lead(II). Based on the similar results obtained for all potentiostats we choose a homemade instrument with a carbon tip working electrode for our subsequent environmental experiments, in which we analyzed maize and sunflower seedlings and rainwater obtained from various sites in the Czech Republic.

## 1. Introduction

Heavy metal ions are natural components of Earth’s crust. Their content in soil varies from very low (femtograms) to high (milligrams). However due to anthropogenic activities their content can be elevated at the site of the action. High concentrations of heavy metal ions can injure human health and pollute the environment. It is a common knowledge that toxic heavy metal ions (lead, cadmium and mercury) are able to enter organisms and interfere with several important metabolic processes. The presence of toxic ions in a plant cell damages homeostasis, transpiration, *etc.* [1]. Plants are capable of surviving this abiotic stress due to a number of protective mechanisms [2–4]. The result is that the plant lives and develops in the polluted environment and, moreover, accumulates the heavy metal ions in its tissues. If such plants are harvested, the foodstuffs derived from them may pose a threat to animal and human health [5,6].

Due to the above-mentioned facts the development of simple analytical instruments, methods and procedures with low detection limits are needed [7]. Analytical methods and instruments for detection of cadmium(II) [8–11] and lead(II) [12–16] ions have been reviewed several times. Electrochemical ones are among the very sensitive analytical methods available for detection of heavy metal ions [17–19]. The classic instrument consists of a potentiostat/galvanostat with an electrochemical cell including three electrodes (working, reference and auxiliary). However the current trend of analytical techniques is to miniaturize the whole instrument due to the many advantages of small devices including portability, low costs and less demands on service and operations, sufficient sensitivity and selectivity [20,21]. As the working electrode, a hanging mercury drop electrode (HMDE) is commonly used [22]. The HMDE can be also modified with biologically active substances to improve the sensitivity or selectivity of heavy metal ion detection [23–26]. Due to the adverse effects of Hg(II) and many restrictions for usage of this metal, carbon electrodes have been promoted as an alternative [27–29]. Moreover, in the miniaturization of whole instruments, carbon electrodes have many advantages compared to HMDE [20,21]. Screen-printed carbon electrodes belong to the most suitable carbon electrodes for *in situ* environmental analysis [30–34]. Besides the electrodes, the potentiostat controlling the electrode system also has to be miniaturized, portable and easy-to-use. The aim of this work was to utilize and compare electrochemical instruments for the easy and sensitive determination of heavy metal ions. The instruments were further employed to analyse real samples.

## 2. Results and Discussion

### 2.1. Automated Electrochemical Detection of Cadmium(II) and Lead(II) Ions at a Hanging Mercury Drop Electrode—Metrohm Potentiostat

**Figure 1.**
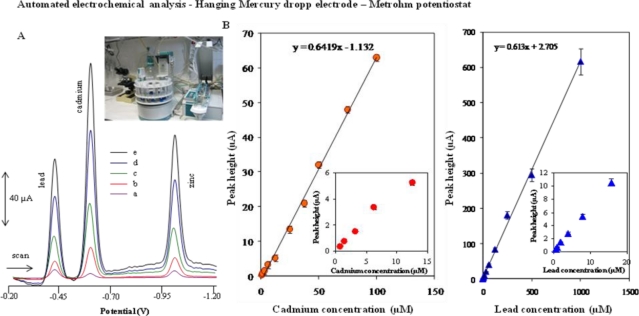
(A) DP voltammograms of lead(II) and cadmium(II) ions: a (Pb^2+^ 10.0 μM, Cd^2+^ 10.0 μM); b (Pb^2+^ 15.6 μM, Cd^2+^ 25.0 μM); c (Pb^2+^ 32.3 μM, Cd^2+^ 100.0 μM); d (Pb^2+^ 62.5 μM, Cd^2+^ 175.0 μM); e (Pb^2+^ 125.0 μM, Cd^2+^ 250.0 μM). (B) The dependence of peak height on concentration of the metals as follows for cadmium (0.75–100 μM) and for lead (0.5–1,000 μM); in insets: for cadmium (0.75–12.5 μM) and for lead (0.5–15.6 μM). Potentiostat: Autolab.

Electrochemical detection of cadmium(II) and lead(II) ions at a mercury working electrode is routinely used. Redox signals for cadmium were observed at −0.6 V and for lead at about −0.4 V *versus* Ag/AgCl 3M KCl. Stripping techniques markedly lowered the detection limits for these ions [35–42]. The metals are preconcentrated by electrodeposition into a small volume mercury electrode. The preconcentration is done by cathodic deposition at a controlled time and potential. The deposition potential is usually 0.3–0.5 V more negative than the standard redox potential for the least easily reduced metal ions to be determined. The metal ions reach the mercury electrode by diffusion and convection, where they are reduced and concentrated as amalgams [43]. Typical DP voltammograms of cadmium(II) and lead(II) ions measured with HMDE using automated electrochemical analyser are shown in Figure 1. The calibration curves were strictly linear with detection limits on the order of hundreds of pM. Relative standard deviation did not exceed 2%.

### 2.2. Electrochemical Detection of Cadmium(II) and Lead(II) Ions—PalmSens potentiostat

Differential pulse anodic stripping voltammetry using HMDE as a working electrode is among the most sensitive analytical techniques used for heavy metal ion detection. However, from a technological point of a view, the non-solid electrodes have much more lower miniaturization potential than solid electrodes, like silver, gold, carbon or platinum. The printing of electrodes is a promising technology for further miniaturization. Screen-printing is an undemanding non-vacuum method for spreading of thixotropic materials. Single layers are created by pressing the paste on the substrate through the screen. The advantage of this technique is its simplicity, high mechanical and electric properties, easy connection to other circuits and particularly, low-cost [44], yet despite the many advantages of printed electrodes, their fabrication requires sophisticated technological equipment including highly professional servicing.

**Figure 2.**
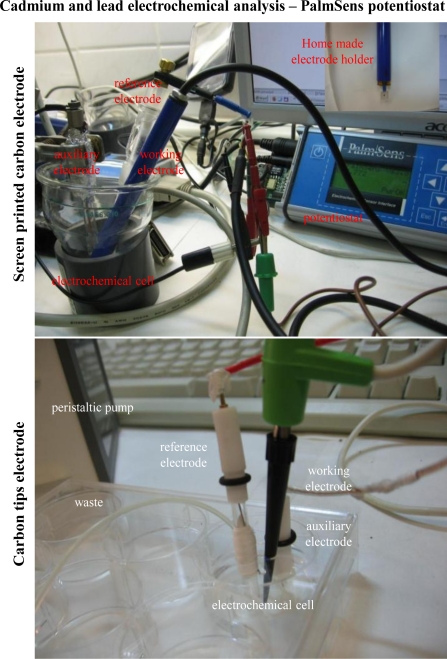
Photos of screen printed carbon electrode and/or carbon tips electrode connected to PalmSens potentiostat.

Based on the aforementioned facts, we tested two miniaturized electrode systems, both connected to a PalmSens potentiostat, for detection of cadmium and lead ions (Figure 2). The first system uses screen-printed carbon electrodes (Figure 2A) and the second commercially available pipette tips (Figure 2B).

**Figure 3.**
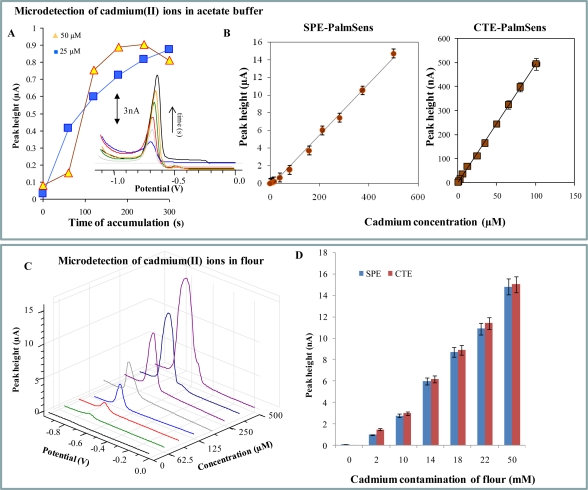
Differential pulse voltammetric detection of cadmium(II) ions at SPE and/or CTE connected to PalmSens potentiostat. (A) The dependence of cadmium(II) ions peak height on accumulation time (concentration of Cd(II) is 20 μM), in inset: typical DP voltammograms of cadmium(II) ions measured at various times of accumulation with CTE. (B, C) Calibration curves. (D) DP voltammograms of various concentrations of cadmium(II) ions measured with SPE. Experimental parameters were as follows: initial potential 0 V, end potential −0.8 V, potential step 5 mV.

A pipette tip (1 ml, Tosoh, Japan) is made from polymeric material and coated by graphite. The presence of such polymeric and carbon-based material enables us to use this accessory as a working electrode, because it is of conductive resin. Based on the mentioned facts, these electrodes can be used for detection of substances undergoing reduction and/or oxidation on the surface of such electrodes without any pre-treatment procedures. We investigated dependence of cadmium(II) ions peak height on time of accumulation of these ions on both working electrodes (SPE—screen-printed electrodes and CTE—carbon tip electrodes). Cadmium(II) ions adsorb well on the surface of SPE and CTE, because the cadmium(II) ion peak was enhanced with the increasing accumulation time (Figure 3A). In the case of SPE the highest signal was determined after a 240 s long accumulation. The cadmium(II) ion peak detected at CTE was enhanced over the whole tested interval (Figure 3A).

Typical DP voltammograms of cadmium(II) ions measured with the carbon tip electrode after various accumulation times are shown in the inset of Figure 3A. Peak potential is −0.64 V and it gradually shifts to more negative potentials with increasing heavy metal concentration. We also determined that the working electrode prepared from a pipette tip provides repeatable electrochemical signals for the heavy metals ions of interest with relative standard deviation (R.S.D.) lower than 9% (n = 5), whereas R.S.D. of measurements with SPE was 3.8%. In spite of the fact that SPE gave results with lower R.S.D., CTE had higher sensitivity (approximately 2×) to cadmium(II) ions. Based on the aforementioned results, we used an accumulation time of 180 s for the measurement of dose-response curves. The obtained curves were strictly linear within a concentration interval from 1 to 500 μM with regression equations y = 0.0292x − 0.3462, R^2^ = 0.9952 (SPE, Figure 3B) and y = 0.029x − 0.410, R^2^ = 0.9950 (CTE, Figure 3C). DP voltammograms of various concentrations of cadmium(II) ions measured with SPE are shown in Figure 3D. Detection limits of cadmium(II) ions estimated by diluting (time of accumulation 180 s) were 50 nM for SPE and 20 nM for CTE.

**Figure 4.**
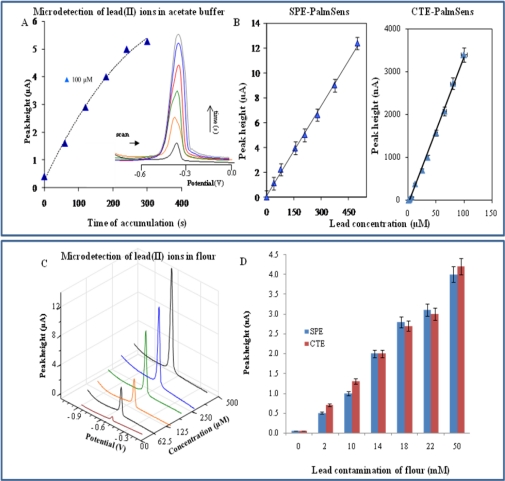
Microdetection of lead(II) ions at carbon tip. (A) The dependence of lead(II) ions peak height on time of accumulation, in inset: typical DP voltammograms of lead(II) ions under various times of accumulation. (B) Calibration curve. (C) DP voltammograms of various concentrations of lead(II) ions. (D) Detection of lead(II) ions in contaminated flour.

Concerning the detection of lead(II) ions at the SPE and CTE, we also measured the dependence of peak height on accumulation time (Figure 4A). The trend was similar to the cadmium-dependences. The lead(II) ions peak measured with SPE was enhanced up to 240 s and then decreased slightly, which could be attributed to saturation of the working electrode or changes in the physico-chemical properties of the electrode due to the long interaction with heavy metal ions. A-CTE-measured-curve was enhanced over the whole tested interval (Figure 4A). Typical voltammograms of lead(II) ions measured with the carbon tip under various times of accumulation are shown in the inset of Figure 4A. The signal potential is −0.43 V and gradually shifts to more negative potentials with increasing heavy metal concentration. Like cadmium(II) ions, the R.S.D. of CTE measurements was higher (9.1%, n = 5) in comparison with SPE (4.2%, n = 5), however, the sensitivity of CTE to lead(II) ions was more than three times higher compared to SPE. Based on the fore-mentioned results, we used accumulation time of 240 s for the measurement of dose-response curves. The obtained curves were strictly linear within concentration interval from 40 to 500 μM with regression equations y = 0.0242x + 0.0656, R^2^ = 0.9981 (SPE, Figure 4B) and y = 0.073x + 0.314, R^2^ = 0.9980 (CTE, Figure 4C). DP voltammograms of various concentrations of lead(II) ions measured with SPE are shown in Figure 4D. Detection limits of lead(II) ions estimated by diluting (time of accumulation 240 s) were 500 nM for SPE and 150 nM for CTE.

### 2.3. Cadmium and Lead Ions Detection by Micropotentiostat

**Figure 5.**
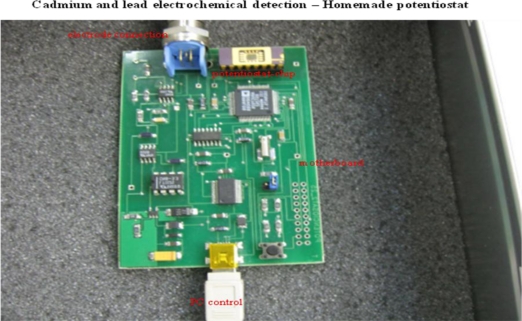
Photo of homemade potentiostat with chip and controlling circuits.

For the next experiments we fabricated a simple micropotentiostat [45]. The apparatus consists of the 10 × 10 cm printed circuit board mounted with SMD devices (Figure 5). The potentiostat on a chip is built into this board. The apparatus is connected to a PC by a USB connector and basic instructions are transmitted into the chip thanks to a control program. Electrodes are connected with the micropotentiostat via a 3-way TRIAD connector.

**Figure 6.**
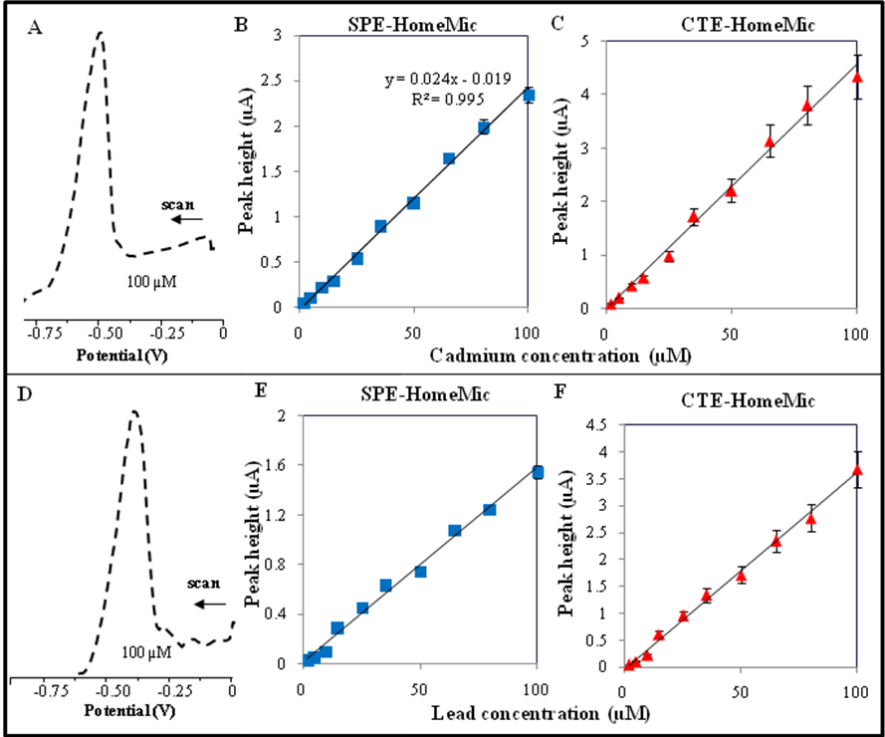
Cadmium(II) ions. (A) DP voltammogram of cadmium(II) ions detected at SPE. Calibration curves measured with (B) SPE and/or (C) CTE. Lead(II) ions. (A) DP voltammogram of lead(II) ions detected at SPE. Calibration curves measured with (B) SPE and/or (C) CTE. Poteniostat: homemade. Experimental parameters were as follows: initial potential 0 V, end potential −0.8 V, potential step 5 mV.

Cadmium(II) and lead(II) ion detection were carried out at SPE and CTE by differential pulse voltammetry in plastic electrochemical cell with low volume of 0.2 M acetate buffer (500 μl) as supporting electrolyte. DP voltammogram of 100 μM cadmium(II) ions measured with SPE is shown in Figure 6A. The potential of cadmium(II) ions peak was −0.65 V. Compared to previous measurements the potential is shifted by more than 50 mV to negative values. This fact is probably caused by the materials used for printing the working electrode. For measuring dose-response curves we choose the previously optimized accumulation time of 180 s. Calibration curves for cadmium(II) ions measured with SPE and/or CTE are shown in Figures 6B and/or 6C, respectively.

Both calibration curves were linear with regression equations y = 0.024x − 0.019, R^2^ = 0.9956 (SPE, Figure 6B) and y = 0.045x − 0.018, R^2^ = 0.9920 (CTE, Figure 6C). Moreover, it clearly follows from the results obtained that a peak height measured with CTE is approximately two times higher compared to SPE. Nevertheless, R.S.D. of CTE’s measurements was again higher (9.5%, n = 5) compared to SPE (3.6%, n = 5). Detection limits of cadmium(II) ions estimated by diluting (time of accumulation 180 s) were 500 nM for SPE and 150 nM for CTE.

The potential of the obtained lead(II) ions peak measured with CPE was app. −0.45 V (Figure 6D). The shift of potential compared to HMDE analyses was approximately same as in case of cadmium(II) ions. In the case of lead(II) ions detection, we used accumulation time of 240 s to measure dose-response curves. Both calibration curves were linear with regression equations y = 0.0158x + 0.0073, R^2^ = 0.992 (SPE, Figure 6B) and y = 0.036x − 0.018, R^2^ = 0.995 (CTE, Figure 6C). A peak height measured with CTE is approximately two times higher compared to SPE. Nevertheless, R.S.D. of CTE’s measurements was again higher (9.1%, n = 5) compared to SPE (3.2%, n = 5). Detection limits of cadmium(II) ions estimated by diluting (time of accumulation 180 s) were 1 μM for SPE and 500 nM for CTE.

### 2.4. Comparison of the Potentiostats and Working Electrodes Used for Detection of Cadmium(II) and Lead(II) Ions

In the previous paragraphs we showed the use of three various electrochemical systems for detection of cadmium(II) and lead(II) ions. The first (Autolab) potentiostat used with HMDE was the most sensitive, with detection limits in the pM range. This instrument, however, is the largest among the tested potentiostats, using HMDE and therefore its potential for usage *in situ* is doubtful. The second tested potentiostat was a readily portable instrument called PalmSens with SPE and/or CTE as working electrodes. The detection limits were down to tens of nM for cadmium(II) ions and hundreds of nM for lead(II) ions. This instrument was less sensitive compare to Autolab but utilizable for *in situ* measurements. The great advantages of this potentiostat are low cost, good portability and the ability to use commonly available pipette tip as working electrode. The sensitivity of this instrument is sufficient for analysis of environmental samples. Our home-made potentiostat connected with SPE and/or CTE was also less sensitive to the heavy metal ions of interest compared to the Autolab with detection limits of hundreds of nM for cadmium(II) and units of μM for lead(II) ions. Much worse sensitivity can be associated with higher noise of the instruments and much lower range of measured currents. Nevertheless, this potentiostat is sensitive enough to evaluate contamination of the environment, because the environmental limits for both metals are higher than the detection limits of this instrument (Table 1).

Further, we tested all used potentiostats and working electrodes for the analysis of environmental samples (rainwater, flour and plant extract) with artificially added cadmium(II) and lead(II). The samples of contaminated flour were weighed and added to acetate buffer. These solutions were vortexed for 15 minutes. Further, samples were centrifuged and the supernatants obtained were analysed by the previously described electrochemical instruments. Rainwater was used without any pretreatment. Preparation of plant tissues is described in the Experimental section. We were able to quantify cadmium(II) and lead(II) ions simultaneously due to their different peaks potentials. Recovery expressed as ratio between found and given concentration of a heavy metal was higher than 80% for all potentiostats (Table 2). This shows that all instruments can be used for analysis of environmental samples. Based on the similar results obtained for all potentiostats we choose the homemade instrument with CTE in our subsequent environmental experiments.

### 2.5. Determination of Cadmium(II) and Lead(II) Ions in Maize and Sunflower Seedlings

**Figure 7.**
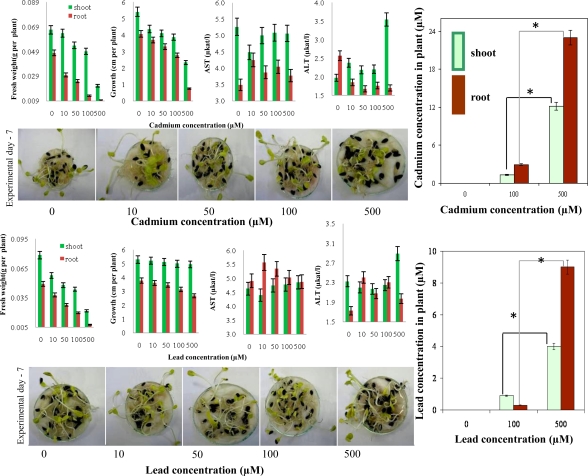
Sunflower seedlings. Changes in fresh weight, growth, AST and ALT activities in sunflower seedlings treated with (A) cadmium(II) ions and/or (B) lead(II) ions. Content of (C) cadmium(II) ions and /or (D) lead(II) ions in shoots and roots of the treated seedlings.

The homemade micropotentiostat was further used to detect cadmium(II) and lead(II) ions in extracts of plants with the aim of monitoring levels of these heavy metals ions in series of biological samples. For this purpose the simple biological experiment was designed. Maize and sunflower kernels were placed onto filter paper. These kernels were treated with cadmium(II) or lead(II) ions (0, 10, 50, 100 and 500 μM) for seven days in dark at 23 °C. Samples were collected at the end of the treatment. Sampled seedlings were carefully rinsed with distilled water and ethylenediaminetetraacetic acid (EDTA) solution, divided into roots and shoots and analysed. Primarily, we aimed our attention at basic growth and biochemical parameters of sunflower seedlings treated with cadmium(II) and lead(II) ions. Both heavy metal ions had an adverse effect on fresh weight and growth of roots and shoots of the seedlings (Figure 7A, B). However, activities of aspartate transaminase (AST) and alanine transaminase were affected differently by cadmium(II) and lead(II) ions. AST activity was higher or similar in plants treated with Cd(II) and/or Pb(II) compared to control plants. Nevertheless, activity of ALT decreased in roots and increased in shoots of plants treated with Cd(II). In plants treated with Pb(II) ALT activity increased in roots and was similar in shoots. The content Cd(II) and/or Pb(II) of in plants treated with 10 and 50 μM was under detection limit of the instrument used (Figure 7C, D). It follows from the results obtained that content of both metal ions enhanced significantly (*p < 0.05) with increasing concentration of a metal and time of the treatment. Higher content was determined in roots compared to shoots in case of both metal ions.

**Figure 8.**
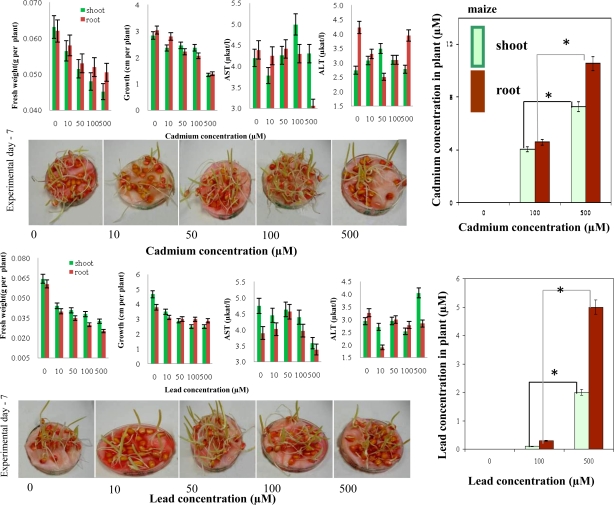
Maize seedlings. Changes in fresh weight, growth, AST and ALT activities in maize seedlings treated with (A) cadmium(II) ions and/or (B) lead(II) ions. Content of (C) cadmium(II) ions and /or (D) lead(II) ions in shoots and roots of the treated seedlings.

Maize seedlings were prepared like the above-mentioned sunflower seedlings. Both heavy metal ions had an adverse effect on fresh weight and growth of roots and shoots of the seedlings (Figure 8A, B). The effect of heavy metal ions on activities of ALT and AST was also negative. In almost all experimental groups the activities decreased or were similar to control plants. The content Cd(II) and/or Pb(II) of in plants treated with 10 and 50 μM was again under the detection limit of the instrument used as in the case of sunflower seedlings (Figure 8C, D). It follows from the results obtained that content of both metal ions enhanced significantly (*p < 0.05) with increasing concentration of a metal and time of the treatment. Higher content was determined in roots compared to shoots in case of both metal ions.

### 2.6. Cadmium(II) and Lead(II) Ions Monitoring in Wetlands

**Figure 9.**
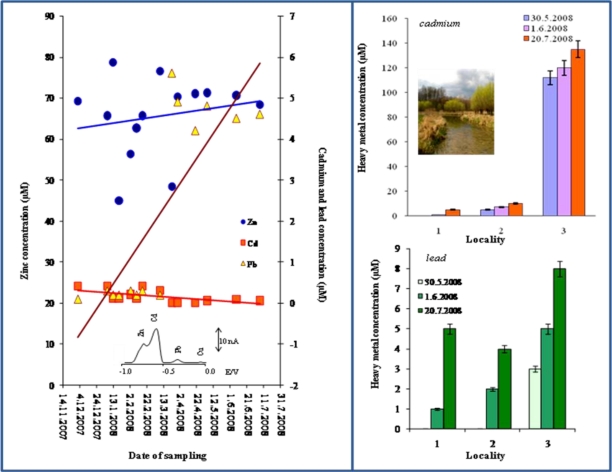
(A) Concentration of zinc(II), cadmium(II) and lead(II) ions in rainwater sampled from 14th November 2007 to 31st July 2008 in Boritov, Czech Republic. Concentration of (B) cadmium(II) ions and/or (C) lead(II) ions in water samples obtained from the Zidlochovice area (southern Moravian region, Czech Republic). The metal ions were determined by using homemade potentiostat and CTE as working electrode.

One of the most important aims of analytical chemistry is the monitoring of heavy metal ions levels in living environment. *In situ* analysis can bring a plenty of otherwise very hardly pursuable information and data. If we automate whole process of measurement, we have at our disposal a tool for on-line monitoring of environmental targets. It is clear that there is great demand on the ability of these instruments to carry out simultaneous analysis of target analysis. We attempted to detect cadmium(II) and lead(II) ions in rainwater (sampling times from 14th November 2007 to 31st July 2008, sampling place: Boritov, Czech Republic). Well developed and separated voltammetric signals at their characteristic potentials (Cd–0.6 V and Pb–0.4 V) were obtained (inset in Figure 9A). Moreover, we detected zinc(II) ions. The total amount of cadmium and lead was determined in units of μM (Figure 9A).

In addition, we analysed water samples obtained from the Zidlochovice area (southern Moravian region, Czech Republic). Three different samples from three different sampling places in the locality were tested. We did not detect some of the monitored heavy metal (Figure 9B, C). In locality 2, we determined the cadmium(II) ions at 10 μM. In locality 3, the cadmium(II) ions concentration was very high and ranged about 110 μM; lead(II) ions were also detected and their content was 5 μM.

## 3. Experimental Section

### 3.1. Chemicals, Materials and pH Measurements

Chemical used was purchased from Sigma Aldrich Chemical Corp. (USA) in ACS purity unless noted otherwise. The stock standard solutions was prepared with ACS water (Sigma-Aldrich, USA) and stored in the dark at −4 °C. Working standard solutions were prepared daily by dilution of the stock solutions. The pH value was measured using WTW inoLab Level 3 with terminal Level 3 (Weilheim, Germany), controlled by the personal computer program (MultiLab Pilot; Weilheim, Germany). The pH-electrode (SenTix-H, pH 0–14/3M KCl) was regularly calibrated by set of WTW buffers (Weilheim, Germany). Deionised water underwent demineralization by reverse osmosis using the instruments Aqua Osmotic 02 (Aqua Osmotic, Tisnov, Czech Republic) and then it was subsequently purified using Millipore RG (Millipore Corp., USA, 18 MΩ–MiliQ water.

### 3.2. Plant Cultivation

Maize (*Zea mays* L.) and sunflower (*Heliantus annuus* L.) were used in our experiments. Maize and sunflower kernels were germinated on wet filter paper in Petri dish in Versatile Environmental Test Chamber (MLR-350 H, Sanyo, Japan) for 7 days in dark at a temperature 23.5–25 °C and humidity 71–78%. Before germination, CdCl_2_ and/or Pb(NO_3_)_2_ were added to Petri dish with kernels in the following concentrations 0, 10, 50, 100 and 500 μM. Kernels germinated without heavy metals additions were used as a control. Seedlings of each concentration were harvested at the end of the treatment (7th day) and their roots were rinsed three times in distilled water and 0.5 M EDTA. In addition, each harvested seedling was divided into shoots and roots.

### 3.3. Sample Preparation

Weighed plant tissues (approximately 0.2 g) were transferred to a test-tube, and liquid nitrogen was added. The samples were frozen to disrupt the cells. The frozen sample was transferred to a mortar and ground for 1 minute. Then, 1,000 μL of 0.2 M phosphate buffer (pH 7.2) was added to the mortar, and the sample was ground for 5 minutes. The homogenate was transferred to a new test-tube. The mixture was homogenised by shaking on a Vortex–2 Genie (Scientific Industries, New York, USA) at 4 °C for 30 minutes. The homogenate was centrifuged (14,000 g) for 30 minutes at 4 °C using a Universal 32 R centrifuge (Hettich-Zentrifugen GmbH, Tuttlingen, Germany). Before the analysis the supernatant was filtered through a membrane filter (0.45 μm Nylon filter disk, Millipore, Billerica, Mass., USA).

### 3.4. Automated Spectrometric Measurements

Spectrometric measurements were carried using an automated chemical analyser BS-200 (Mindray, China). Reagents and samples were placed on cooled sample holder (4 °C) and automatically pipetted directly into plastic cuvettes. Incubation proceeded at 37 °C. Mixture was consequently stirred. The washing steps by distilled water (18 mΩ) were done in the midst of the pipetting. Apparatus was operated using software BS-200 (Mindray, China) [46,47].

#### 3.4.1. Determination of ALT (AST) activity

We pipetted 250 μL of substrate consisting of 0.2 M DL-α-alanine (L-aspartate) and 2 mM 2-oxoglutarate in 0.1 M phosphate buffer (pH 7.4) at 37 °C to 50 μl of plant substrate in a plastic microtube. The resulting mixture was incubated at 37 °C for 60 minutes. After that 250 μL of 2,4-dinitrophenylhydrazine in 1 M hydrochloric acid was added to the mixture. The microtube was carefully stirred and loaded into an automatic biochemical analyzer BS-200 (Mindray, China). Experimental parameters of BS-200 were as follows: 30 μL of the previously prepared mixture was pipitted to cuvette and incubated at 37 °C for 20 minutes. Further, 180 μL of 1 M NaOH was added. Ten minutes later, the absorbance was measured at 510 nm.

### 3.5. Autolab

Differential pulse voltammetric measurements were performed with 747 VA Stand instrument connected to 746 VA Trace Analyzer and 695 Autosampler (Metrohm, Switzerland), using a standard cell with three electrodes. The three-electrode system consisted of hanging mercury drop electrode (HMDE) as working electrode, an Ag/AgCl/3 M KCl reference electrode and a glassy carbon auxiliary electrode. For smoothing and baseline correction the software GPES 4.9 supplied by EcoChemie was employed. Acetate buffer (0.2 M CH_3_COOH + 0.2 M CH_3_COONa) was used as the supporting electrolyte. The measurements were performed at room temperature.

### 3.6. PalmSens

Differential pulse voltammetric measurements were performed with PalmSens instrument connected to PC (PalmSens, The Netherlands), using a miniaturised cell with three electrodes. The three-electrode system consisted of SPE and/or CTE as working electrode, an miniaturized Ag/AgCl/3 M KCl reference electrode (CH Instruments, USA) and a platinum wire auxiliary electrode. For smoothing and baseline correction the PalmSens software supplied by PalmSens was employed. The supporting electrolytes as acetate buffer (0.2 M CH_3_COOH + 0.2 M CH_3_COONa) were used according to Vacek *et al.* [48]. The measurements were carried in low volume plastic cell (1 mL of supporting electrolyte) at room temperature.

### 3.7. Homemade Potentiostat

The potenciostat was designed at Brno University of Technology, Czech Republic. The chip was designed using AMIS CMOS 0.7 μm technology and fabricated under the Europractice program. Memory cells of 48 bytes were implemented with the potentiostat using VERILOG [45]. The integrated measurement system has two sections. Analogue section is designed for regulation of the potential and for measuring current (1 nA to 10 mA). Digital part includes PROM memory, control logic, multiplexers and shift registers. Specification of the type, series and calibration data of the sensor are stored in the PROM of the chip. This apparatus consists of basic plate on which the connector TX721 1115 with 2.54 mm pin spacing and the connector 0039532035 from the manufacturer Molex with pin spacing 1.25 mm are placed. The whole microchip is powered by 5 V. The designed analogue ground is at potential of 2.5 V inside the microchip which provides ±2.5 V to supply the analogue part (Figure 5). The instrument is controlled by the computer program for cyclic and differential pulse voltammetry. The three-electrode system consisted of SPE and/or CTE as working electrode, an Ag/AgCl/3 M KCl reference electrode (Metrohm, Switzerland) and carbon auxiliary electrode. Measurements were carried out with the same standard cell as for Autolab potentiostat. The supporting electrolytes as acetate buffer (0.2 M CH_3_COOH + 0.2 M CH_3_COONa) were used. The measurements were performed at room temperature.

### 3.8. Fabrication of Screen Printed Electrodes

A screen-printed working electrode was fabricated using standard thick-film cermet paste on an alumina substrate with dimension 25.4 × 7.2 mm. Paste used for leads and contact pads was AgPdPt based paste type ESL 9562-G (ESL Electroscience, UK). The working electrode was fabricated from carbon paste BQ-221 (DuPont, USA). Isolation has been made from dielectric paste ESL 4913-G (ESL Electroscience, UK).

### 3.9. Biotope Knizeci Forest and Rainfall Water

Wetland biotope originated during recultivation interventions nearby Nosislav (Czech Republic) in 1998. The samples of water were collected in habitat to monitor heavy metal content. Rainfall water was sampled in Boritov, Czech Republic.

### 3.10. Descriptive Statistics

Data were processed using MICROSOFT EXCEL® (USA) and STATISTICA.CZ Version 8.0 (Czech Republic). Results are expressed as mean ± standard deviation (S.D.) unless noted otherwise (EXCEL®). Statistical significances of the differences were determined using STATISTICA.CZ. Differences with p < 0.05 were considered significant and were determined by using of one way ANOVA test (particularly Scheffe test), which was applied for means comparison.

## 4. Conclusions

In spite of the fact the capability and sensitivity of analytical instrumentation have increased in recent years, the routine determination of trace metals in complex media, such as lake water, is still difficult [49]. There are at least two challenging areas in trace metal analysis, being the speciation of inorganic and organic forms; and the trace and ultra trace determination in complex matrices, such as biological fluids and tissues, environmental materials and oils. For these purposes low cost and readily portable sensors and biosensors can be used [23–26,50–60]. In the present study we report on the use of several electrochemical instruments to detect cadmium(II) and lead(II) ions. Considering the fact that we used a homemade micropotentiostat and carbon tips as a working electrode, we proposed an easy-to-use and low cost apparatus still sensitive enough to analyse real samples such as tissues of plants and waters.

## Figures and Tables

**Table 1.** Detection of cadmium and lead—analytical parameters.

**Element**	**Potential (V)**	**Calibration curve equations (nA)— HMDE**	**R^2^**	**Linear dynamic range (μM)**	**LOD (nM)**	**LOC (μM)**	**Relative standard deviation (%)**
							
Cd ^a,b^	−0.595	I = 0.6419c − 1.132	0.9970	0.2–150	100	0.25	1.5
Pb ^a,b^	−0.405	I = 0.6138c + 2.7051	0.9976	2.5–1,000	500	1.0	1.9

**Element**	**Potential (V)**	**Calibration curve equations (nA) — SPE-PalmSens**	**R^2^**	**Linear dynamic range (μM)**	**LOD (nM)**	**LOC (μM)**	**Relative standard deviation (%)**
							
Cd ^a,b^	−0.725	I = 4.955c − 0.713	0.9980	0.5–100	100	0.3	2.5
Pb ^a,b^	−0.455	I = 34.37c − 93.81	0.9969	6–100	500	1.1	2.0

**Element**	**Potential (V)**	**Calibration curve equations (μA) — CTE-PalmSens**	**R^2^**	**Linear dynamic range (μM)**	**LOD (nM)**	**LOC (μM)**	**Relative standard deviation (%)**
							
Cd ^a,b^	−0.640	I = 0.0292c − 0.3462	0.9952	1–500	50	0.1	3.8
Pb ^a,b^	−0.455	I = 0.0242c + 0.0656	0.9981	5–500	500	1.0	4.3

**Element**	**Potential (V)**	**Calibration curve equations (nA) — SPE-HomeMic**	**R^2^**	**Linear dynamic range (μM)**	**LOD (nM)**	**LOC (μM)**	**Relative standard deviation (%)**
							
Cd ^a,b^	−0.663	I = 24.41c − 18.13	0.9953	2–100	500	1.0	5.8
Pb ^a,b^	−0.453	I = 15.68c + 12.11	0.9902	5–100	800	1.2	6.9

**Element**	**Potential (V)**	**Calibration curve equations (nA) — CTE-HomeMic**	**R^2^**	**Linear dynamic range (μM)**	**LOD (nM)**	**LOC (μM)**	**Relative standard deviation (%)**
							
Cd ^a,b^	−0.685	I = 2.20c − 1.99	0.9900	2–100	650	1.2	8.8
Pb ^a,b^	−0.457	I = 1.53c + 1.39	0.9922	5–100	880	1.8	7.3

a ..... time of accumulation 120 s at accumulation potential −1 V

b ..... number of measurement (n = 6)

I..... peak current

c .... element concentration

**Table 2.** Comparison of detection of cadmium(II) and lead(II) ions artificially added to various types of samples (rainwater, flour and plant extract, n = 5).

**Metal (samples)**	**Added (μM)**	**SPE-PalSens (μM)**	**SPE-HomeMic (μM)**	**CTE-PalmSens (μM)**	**CTE-HomeMic (μM)**	**HMDE (μM)**
Cd ^a^	50	49.5 ± 5.5	52.5 ± 10.5	50.8 ± 7.5	53.5 ± 11.8	50.5 ± 3.5
Pb ^a^	50	50.4 ± 6.6	54.0 ± 12.0	50.5 ± 4.5	55.5 ± 10.9	50.3 ± 4.5
Cd ^a^	10	9.5 ± 6.5	10.5 ± 8.8	10.3 ± 7.5	10.0 ± 6.8	10.5 ± 3.3
Pb ^a^	10	9.9 ± 5.9	11.0 ± 9.3	10.8 ± 8.5	10.5 ± 10.8	10.3 ± 4.5
Cd, Pb ^a^	5	4.7 ± 0.8/4.8 ± 0.6	4.9 ± 0.5/4.8 ±0.4	5.3. ± 0.4/4.8 ± 0.4	5.2. ± 0.3/4.9 ± 0.3	5.0. ± 0.2/4.9 ± 0.3
Cd, Pb in rainwater ^b^	5	4.8 ± 0.7/4.9 ± 0.8	5.1 ± 0.6/4.9 ± 0.6	5.5. ± 0.7/5.3 ± 0.5	5.3. ± 0.3/5.0 ± 0.2	5.0. ± 0.1/4.9 ± 0.3
Cd, Pb in flour ^c^	5	4.4 ± 0.8/4.5 ± 0.8	4.3 ± 0.7/4.6 ± 0.7	4.5. ± 0.7/4.6 ± 0.5	4.7. ± 0.6/4.6 ± 0.6	4.7. ± 0.5/4.6 ± 0.7
Cd, Pb in plant ^d^	5	4.1 ± 0.9/4.7 ± 0.8	4.6 ± 0.9/4.8 ± 0.9	4.5. ± 0.7/4.6 ± 0.7	4.5. ± 0.9/4.6 ± 0.8	4.6. ± 0.6/4.7 ± 0.7

a ...samples in 0.2 M acetate buffer, pH 5.0

b ...1.7 ml rain wather in 0.2 M acetate buffer, pH 5.0

c ....0.1 g flour extract in 0.2 M acetate buffer, pH 5.0

d ...0.1 g plant extract in 0.2 M acetate buffer, pH 5.0

SPE-PalmSens ... Screen Printed Electrode connected with PalmSens micropotentiostat

SPE-HomeMic ... Screen Printed Electrode connected with Homemade micropotentiostat

CTE-PalmSens ... Carbon Tips Electrode connected with PalmSens micropotentiostat

CTE-HomeMic ... Carbon Tips Electrode connected with Homemade micropotentiostat

HMDE ... Hanging Mercury Drop Electrode connected with Autolab potentiostat
